# A facile colorimetric method for the quantification of labile iron pool and total iron in cells and tissue specimens

**DOI:** 10.1038/s41598-021-85387-z

**Published:** 2021-03-16

**Authors:** Usama Abbasi, Srinivas Abbina, Arshdeep Gill, Vriti Bhagat, Jayachandran N. Kizhakkedathu

**Affiliations:** 1 Department of Pathology and Laboratory Medicine, The University of British Columbia, Vancouver, BC Canada; 2 Centre for Blood Research, Life Sciences Institute, The University of British Columbia, Vancouver, BC Canada; 3 Department of Chemistry, The University of British Columbia, Vancouver, BC Canada; 4 The School of Biomedical Engineering, The University of British Columbia, Vancouver, BC Canada

**Keywords:** Iron, Analytical biochemistry, Chemical biology

## Abstract

Quantification of iron is an important step to assess the iron burden in patients suffering from iron overload diseases, as well as tremendous value in understanding the underlying role of iron in the pathophysiology of these diseases. Current iron determination of total or labile iron, requires extensive sample handling and specialized instruments, whilst being time consuming and laborious. Moreover, there is minimal to no overlap between total iron and labile iron quantification methodologies—i.e. requiring entirely separate protocols, techniques and instruments. Herein, we report a unified-ferene (*u*-ferene) assay that enables a 2-in-1 quantification of both labile and total iron from the same preparation of a biological specimen. We demonstrate that labile iron concentrations determined from the *u*-ferene assay is in agreement with confocal laser scanning microscopy techniques employed within the literature. Further, this assay offers the same sensitivity as the current gold standard, inductively coupled plasma mass spectrometry (ICP-MS), for total iron measurements. The new *u*-ferene assay will have tremendous value for the wider scientific community as it offers an economic and readily accessible method for convenient 2-in-1 measurement of total and labile iron from biological samples, whilst maintaining the precision and sensitivity, as compared to ICP-MS.

## Introduction

Iron is an essential element for almost all living organisms; it serves as a prosthetic group for a variety of proteins, due to its high redox potential, involved in vital cellular processes, including energy metabolism, oxygen transport, detoxification of reactive oxygen species, and DNA synthesis^1–3^. Due to this, not only iron homeostasis but also iron redox-state homeostasis is strictly regulated^4,5^. Iron homeostasis is tightly regulated by dietary absorption, and undergoes a dynamic cycle of recycling and utilization^6^. Any changes in iron homeostasis can lead to conditions including iron overload diseases (e.g. thalassemia, hemochromatosis) or iron deficiency causing cognitive defects in children^7^. Excess cellular iron is shown to induce liver cirrhosis, cardiomyopathy and diabetes mellitus^8,9^. Localized iron accumulation is often noted in neurodegenerative diseases, including Friedreich’s Ataxia, Parkinson’s, Alzheimer’s disease, and Huntington disease^8–10^.

Our current understanding points to the fact that the pathogenesis of iron-induced diseases is attributed to the combination of increased iron along with prolonged exposure to the redox active labile iron pool (LIP)^3,11,12^. Consequently, the accurate, convenient and economic quantification of biological iron—both as *total iron* and *labile iron*—is of tremendous value to assess the iron burden in clinics as well as to study the biology of these disease conditions in academia.

Currently used approaches to quantify *total iron* in biological samples are atomic absorption spectroscopy (AAS)^13–17^, inductively coupled plasma mass spectrometry (ICP-MS)^18–21^, electron paramagnetic resonance spectroscopy^22,23^, and colorimetric methods^17,18,21,24–29^. Both ICP-MS and AAS are highly sensitive, with ICP-MS enabling iron detection as low as parts per trillion. With the exception of colorimetric methods, all other techniques require special instrumentations, extensive sample manipulation and laborious methodologies. The quantification of *labile iron pool* has been reviewed elsewhere^30^ and relies on fluorescent sensors, with an iron binding moiety, that can be investigated via confocal scanning laser microscopy or flow cytometry^11,12,31–44^. The fluorescent-based approaches offer a methodically defined quantification of labile iron which is often difficult to emulate under different circumstances^31,45–47^. Further, these redox active iron (Fe^2+^) probes suffer from lack of metal ion selectivity^48,49^, need of additional chelators and specific reaction conditions such as acidic solution, indirect measurements of iron based on the free radical formation^50^, irreversibility of sensing mechanism, and unwanted quenching of fluorescence in the presence of other metal ions^51^.

As such, robust and easily accessible methods are required to measure iron levels, both *labile* and *total iron*, in different biological specimens including both in vitro and in vivo contexts. Colorimetric methods are great alternatives and have been used since the 1970s, however, this approach is still underdeveloped. Several groups have made progress towards more accurate determination of iron^17,21,29^. However, most of these methods are handicapped by their application only to total iron, typically in vitro conditions. Thus, there is a need to develop a convenient and economical benchtop assay that can quantitatively measure *labile* and *total iron* content from a wide variety of biological specimens. Further, an assay to differentiate and quantify redox active iron would profoundly impact the field.

Herein, we report of a unified-ferene assay (*u*-ferene assay) that enables a 2-in-1 quantification of both labile iron and total iron in biological samples ranging from cultured cells to more complex specimens, including organs, feces and urine, at the same level of precision ICP-MS.

## Materials and methods

Ethics approval was obtained from the University of British Columbia (UBC) for experiments conducted at the Center for Blood Research. All ethics approval for animal studies were obtained from UBC and complied with the Canadian Council of Animal Care guidelines and animal care protocol (A18-0276). All animal studies were carried out at the Experimental Therapeutics laboratory, B.C. Cancer Research Centre, Vancouver, BC, Canada.

### Materials

Eagle’s Minimum Essential Media (EMEM), Dulbecco’s Phosphate Buffered Saline (PBS), Pierce RIPA Lysis Buffer, bovine serum albumin (BSA) standards and Pierce Coomassie (also referred to as Bradford) protein assay kit was purchased from Thermo Fisher Scientific. Deferoxamine mesylate, fetal bovine serum, ferric ammonium citrate (FAC), ammonium acetate, sodium L-ascorbate, ferene (3-(2-Pyridyl)-5,6-di(2-furyl)-1,2,4-triazine-5′,5′′-disulfonic acid disodium salt), ICP-MS grade iron standards, and concentrated nitric acid were obtained from Sigma-Aldrich unless otherwise mentioned Fast-prep tubes (2 mL) were purchased from MP Biomedicals, LLC-Fisher. ICP-MS grade concentrated nitric acid was acquired from VWR.

### Buffers and standards

#### Preparation of ammonium acetate buffer

Ammonium acetate buffer (pH 4.5, 2.5 M) was prepared using ammonium acetate and glacial acetic acid.

#### Preparation of iron standards

Iron standards were prepared from ferric chloride in 2% nitric acid, ranging from 0 to 1000 μM. These standards are prepared by mass; mass of ferric chloride and mass of 2% nitric acid are taken into account to determine the concentrations.

### Cell culture and treatments

#### Iron loading cells

Hepatocarcinoma cell line, HepG2 (ATCC HB-8065) cells with passage numbers between 4 to 9, were seeded at 500,000 cells per well in a 12 well-plate and grown for 2 days. Cells were then loaded with iron; cells were treated with media containing 200 μM iron from ferric ammonium citrate (18 mol% iron) each day for 2 days. After 2 days of iron loading, cells were maintained for an additional day in media without iron. Prior to any analysis, cells were washed with 2–3 times PBS.

#### Cell lysate preparation

HepG2 cells were scraped, pelleted and washed with PBS thrice at 500 g for 5 min. The supernatant was aspirated and cells were lysed in at least 300 μL Pierce RIPA buffer (Thermo Fisher Scientific) with intermittent sonication for 20 min. After lysis, cell debris was pelleted by centrifuging at 21,000 *g* for 10 min. The supernatant was quantitatively transferred into a clean Eppendorf tube for further analysis.

#### Protein measurement

Protein content in cell lysates were measured using the Bradford assay, following manufacturer’s protocol. In brief, working solution was prepared using reagent A and B at a 50:1 ratio, respectively. BSA standards (10 μL) and cell lysates (10 μL) were added into a 96 well plate. Then, 200 μL of Bradford working solution were added to each well. This was kept at 37 °C for 30 min, then cooled to room temperature for 5 min. Absorbance was read at 562 nm. Protein concentrations in cell lysates were interpolated using the standard curve generated.

### Iron overload mouse model

All the animal studies were carried out at the Experimental Therapeutics laboratory, B.C. Cancer Research Centre, Vancouver, BC, Canada. All the protocols were approved by the Institutional Animal Care Committee (IACC), UBC.

C57BL/6 mice were iron overloaded with five repeat doses of Fe-Dextran at 300 mg/kg^52,53^. Mice were administered with Fe-dextran for every 2 days intravenously (Day 1, 3, 5, 8, and 10). Feces and urine were collected in the last 2 days of the study. Mice were euthanized via CO_2_ asphyxiation (on Day 17) and 200 μL whole blood in EDTA & 150 μL plasma was collected. In addition, entire organs were collected following standard procedure; once removed, they were rinsed with PBS, weighed and frozen in liquid nitrogen and stored at –80 °C. Mouse samples are collectively referred to as biological samples.

### Acid digestion of cell lysates and biological samples

Labile iron measurements do not require nitric acid digestion of biological samples, whereas total iron measurements require this acid digestion to release iron from all stores and proteins.

For iron analysis, mice organs were weighed in Fast-Prep tubes and homogenized in 0.5 mL water (BioSpec Product 96+, Bartlesville, OK). Cell lysates (typically 200 μL, unless specified otherwise) and biological samples were transferred into acid-washed glass vials and dried at 100 °C to 120 °C. The evaporation of water hastens the digestion of organic samples with nitric acid. Then, all biological samples were digested with concentrated nitric acid (HNO_3_)—samples were maintained at 100 °C to 120 °C and small volumes (0.5 to 1.0 mL) of concentrated nitric acid were repeatedly added over the course of 5 days. All digested samples were dried at 120 °C, then cooled to room temperature and weighed.

All the required dilutions were performed by mass. Acid-digested biological samples were resuspended in 4% HNO_3_ and weighed again; cell lysates and plasma samples were resuspended in 200 μL, and biological samples (organ homogenates, blood, urine and feces) were resuspended in 1.0 mL. All biological samples were further diluted in 2% HNO_3_ for the quantification of iron, as outlined in the Supplementary Table 1. HNO_3_ (2%) is used for final dilutions for the longevity of vacuum pumps required by ICP-MS.

### Absorbance measurements

For ferene-based iron detection, all mixtures of samples and standards with ferene-containing working solution were spun at 15,000 *g* for 5 min. Then, 200 μL were transferred into a 96 well plate for absorbance measurements. Absorbance was recorded at 595 nm using a SpectraMax 190 microplate reader from Molecular Devices. Iron concentrations were interpolated from the standard curve generated from the iron standards. These concentrations were normalized to the amount of protein analyzed (i.e., nmole of iron per mg of protein).

### The development of the ferene-based iron assay

A modified ferene assay was developed to analyze the labile and total iron content by manipulating ascorbic acid concentration. This section outlines three subsequent methods for the development of this assay, (i) determining iron concentrations in buffer conditions, (ii) distinguishing total and labile iron in cell lysates, and (iii) distinguishing labile and chelatable iron in cell lysates.

#### Determining iron concentrations in buffer conditions

##### Preparation of analytes and working solution

Three analytes were prepared with desired concentrations in distilled water;

- (Fe) 100 μM of free iron.
- (DFO-Fe) 100 μM of iron pre-chelated with 2 mM deferoxamine. 100 μM of iron solution in distilled water was incubated with 2 mM DFO for 48 h.
- (DFO) 2 mM free deferoxamine.

Nine working solutions were prepared, each with 5 mM ferene in ammonium acetate buffer (pH 4.5, 2.5 M) with varying concentration of ascorbic acid (0, 1, 5, 10, 25, 50 100, 250, and 1000 mM). All the solutions were filtered (0.2 μm PVDF syringe filter) before use.

##### Determination of iron concentration

Iron content of the three analytes was measured in nine different sets of working solutions. A fresh calibration curve was generated using iron standards ranging from 0 to 400 μM (as outlined in 2.2.) for each set of working solution. This assay has been conveniently outlined in Supplementary Table 2.

Each set has 100 μL of analyte (either Fe, DFO-Fe, or DFO) and iron standards (eight samples) in separate Eppendorf tubes. To each Eppendorf tube, 100 μL of ammonium acetate buffer (pH 4.5, 2.5 M) and 120 μL working solution were added. The resultant solution was vortexed and left overnight at room temperature. Absorbance was measured as described earlier in “Absorbance measurements”.

Iron concentrations in different analytes in the presence of varying ascorbic acid concentrations were determined by interpolating from the standard curve generated using iron standards (Supplementary Fig. S1). Calibration curves were generated for nine different ascorbic acid concentrations.

#### Distinguishing total and labile iron in iron overloaded HepG2 cell lysates

Iron overload HepG2 cell lysates were prepared as outlined previously. We define labile iron as iron chelated and detected by ferene using a working solution with a specific low ascorbic concentration (10 mM for final assay-see “Labile iron measurements using the u-ferene assay”) from undigested samples. Similarly, total iron is defined as iron chelated and detected by ferene using a working solution with a particular high ascorbic concentration (1 M for final assay-see “Total iron measurements using the u-ferene assay”) from nitric acid digested samples. In order to distinguish labile and total iron, different working solutions with varying ascorbic acid concentrations were investigated.

##### Measurement of ferene-bound iron

A set of four working solutions were prepared, each with 5 mM ferene in ammonium acetate buffer (pH 4.5, 2.5 M) with four varying ascorbic acid concentrations (10, 50, 250, and 1000 mM). For labile iron measurements, 100 μL of as made cell lysates were transferred into separate Eppendorf tubes. For total iron measurements, 100 μL of nitric acid digested cell lysates (as outlined in 2.5) were aliquoted into separate Eppendorf tubes. Iron standards (100 μL) were also aliquoted into separate Eppendorf tubes.

Ammonium acetate buffer (pH 4.5, 2.5 M) (100 μL) and 120 μL of working solution were added to all tubes. The samples were vortexed and left overnight at room temperature. The absorbance was measured in 200 μL of the resultant solution, as outlined in “Absorbance measurements”.

#### Distinguishing labile and chelatable iron in cell lysates

Cell lysates for both non-iron treated and iron overloaded HepG2 cells were prepared as outlined in “Cell culture and treatments”. For the purpose of this investigation, chelatable iron is defined as the portion of labile iron that is chelated by iron chelators and subsequently prevents the ferene-based detection of iron using a working solution at a particular low concentration of ascorbic acid.

##### Chelator treatment of iron overloaded cell lysates

Deferoxamine (DFO), deferiprone (DFP), and deferasirox (DFX) (50 μM each) were prepared in PBS. Iron overloaded cell lysates were treated with chelators; 250 μL of iron loaded HepG2 cell lysates were transferred into a clean Eppendorf tube followed by 50 μL of 50 μM chelator—either DFO, DFP or DFX. As negative controls, 250 μL of non-iron loaded and 250 μL iron loaded HepG2 cell lysates were also transferred into clean Eppendorf tubes followed by 50 μL of PBS only. These samples were left overnight at room temperature.

##### Measurement of ferene-based iron

For labile iron: Working solution was prepared with 5 mM ferene in ammonium acetate buffer (pH 4.5, 2.5 M) with 10 mM of ascorbic acid. Cell lysates and iron standards (100 μL each) were transferred into separate Eppendorf tubes. 100 μL of ammonium acetate buffer (pH 4.5, 2.5 M) and 120 μL of working solution were added to each tube.

For total iron: Working solution was prepared with 5 mM ferene in ammonium acetate buffer (pH 4.5, 2.5 M) with 1 M of ascorbic acid. Acid digested cell lysates (100 μL) (as outlined in 2.5) and iron standards were transferred into separate Eppendorf tubes. Ammonium acetate buffer (100 μL) (pH 4.5, 2.5 M) and 120 μL working solution were added to each tube.

For iron quantification: These tubes were vortexed and left overnight. Absorbance was measured as described earlier in “Absorbance measurements”.

### The unified-ferene assay (*u*-ferene assay): the finalized protocol

A *unified*-ferene (*u*-ferene) assay was developed to enable a 2-in-1 quantification of both labile iron and total iron in a wide variety of biological samples. In this assay, labile and total iron were defined by their experimental parameters; *labile iron* was defined as iron detected in undigested biological samples using 10 mM ascorbic acid in the working solution, and *total iron* was defined as iron detected in nitric acid digested biological samples using 1 M ascorbic acid in the working solution.

This assay has five components, (1) working solution, (2) ammonium acetate buffer (pH 4.5, 2.5 M), (3) iron standards, (4) the sample of interest (either cell lysates or biological mice samples), and (5) absorbance measurements.  “Labile iron measurements using the u-ferene assay” outlines the measurement of labile iron and “Total iron measurements using the u-ferene assay”. outlines the measurement of total iron.

#### The working solutions:

The working solution is composed of ferene (5 mM) and ascorbic acid (either 10 mM for labile iron measurements, or 1 M for total iron measurements) in an ammonium acetate buffer. This is summarized in the Supplementary Table 3. The volume of working solution is determined by a 1:6 molar ratio between iron in the highest standard to ferene at 5 mM in working solution—i.e. when using 100 μL samples, working solution is calculated such that there is 6 times more moles of ferene than the moles of iron present in 100 μL of 1000 μM iron standard. Hence, this assay is applicable for investigations requiring larger sample volumes.

#### Ammonium acetate buffer (see also “Buffers and standards”)

Ammonium acetate buffer was added at a 1:1 volume equivalence to all biological samples and iron standards, i.e. when using 100 μL samples, 100 μL of buffer was added.

#### Iron standards (see also “Buffers and standards”)

When using iron standards for *u*-ferene assay, 1000 μM was used at the highest iron standard, however, this was not considered due to SpectraMax 190 microplate reader’s limit of detection. Iron standards were prepared using FeCl_3_ because this is a primary standard used in ICP-MS. The iron standard curve was compared to another primary source, FeSO_4_, as well to further validate the labile iron measurements and total iron measurements using the *u*-ferene assay (Supplementary Fig. S5).

#### Sample of interest (cell lysates and/or biological samples) (see also “Cell culture and treatments” to “Acid digestion of cell lysates and biological samples”)

Different samples are used. Samples of interest can vary in specimen and complexity, including cell culture lysates to homogenates from mouse tissue samples. It is important to highlight that for labile iron measurements, samples do not require nitric acid digestion, whereas total iron measurements require this digestion to release iron from all stores and proteins.

#### Absorbance measurements

Details are given in “Absorbance measurements”.

#### Labile iron measurements using the u-ferene assay

Labile iron concentrations were determined in cell lysates and plasma samples. Samples (either cell lysates or plasma samples) (100 μL) and iron standards (100 μL) with concentrations ranging from 0 to 1000 μM were transferred into different clean Eppendorf tubes. Ammonium acetate buffer (pH 4.5, 2.5 M) (100 μL) and labile iron working solution (5 mM ferene and 10 mM ascorbic acid prepared in ammonium acetate buffer pH 4.5, 2.5 M) (120 μL) were added to all Eppendorf tubes. This mixture was vortexed and left overnight at room temperature. Absorbance was measured as described earlier in “Absorbance measurements”.

#### Total iron measurements using the u-ferene assay

Total iron concentrations were determined in cell lysates, plasma samples, organ homogenates, blood, urine, and feces. The nitric acid digested samples after their final dilution (200 μL) and iron standards (200 μL) ranging from 0 to 1000 μM were transferred into different clean Eppendorf tubes. 200 μL of ammonium acetate buffer (pH 4.5, 2.5 M) and 240 μL of total iron working solution (5 mM ferene and 1 M ascorbic acid prepared in ammonium acetate buffer pH 4.5, 2.5 M) were added to all Eppendorf tubes. This mixture was vortexed and left overnight at room temperature. Absorbance was measured as described in “Absorbance measurements”.

### Validation of total iron concentrations using inductively coupled plasma mass spectrometry

Biological samples were nitric acid-digested and resuspended in 4% HNO_3_, as described previously. These samples were then appropriately diluted in 2% HNO_3_ with 10 ppb indium (Sigma Aldrich) as the internal standard, as outlined in Supplementary Table 1. Iron standards were also prepared in 2% HNO_3_ with 10 ppb indium, ranging from 0 to 400 ppb, to generate a standard curve. Iron counts were measured on Agilent 7700 series ICP-MS and total iron concentrations were determined from the standard curve.

### Calcein-based labile iron

Intracellular labile iron was measured using Calcein acetoxymethyl ester (Cal-AM), described elsewhere^54^. This is a non-fluorescent dye that becomes fluorescent after enzymatic modification once it permeates the cell membrane^55,56^. This fluorophore binds iron stoichiometrically, which quenches its green fluorescence^55,56^. In short, cells were washed, trypsinized and pelleted at 500 g for 5 min. Then, the cells were resuspended in PBS with 0.2 μM of Cal-AM for 20 min at room temperature. Cellular calcein fluorescence was measured using Beckman Coulter’s flow cytometry. At least 10,000 cells were analyzed and fluorescence was measured by the 488 nm laser and the FITC emission filter (530/20 nm).

### Statistical analysis

All data were performed in at least independent triplicates and presented with error bars that correspond to standard deviations. Technical replicates were also performed, but only independent triplicates were used for statistical analysis. All statistical analyses were performed using GraphPad Prism. The statistical tests and appropriate corrections were outlined in the figure legends.

## Results

### The role of ascorbic acid in iron content measurement by the ferene assay

**Figure 1:**
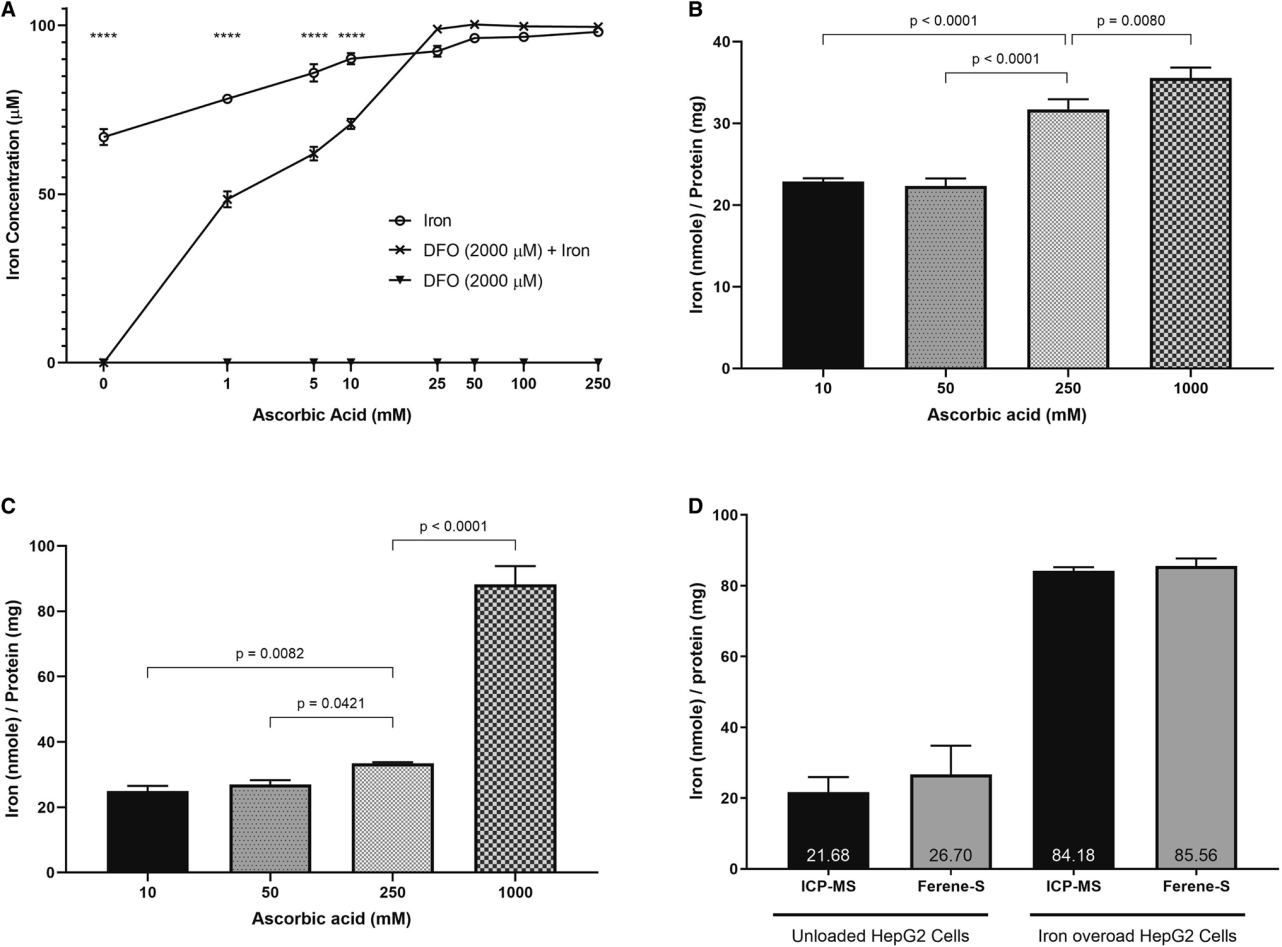
The effect of changing the working solution’s ascorbic acid concentration on the measurement of ferene-chelatable iron. (**A**) Changes in OD 595 nm were measured for three analytes, 100 μM of iron, either free or pre-chelated with 2000 μM deferoxamine (DFO), when changing ascorbic acid. A concentration dependent increase in iron concentration was observed for iron pre-chelated with DFO. A two-way ANOVA was performed with Dunnett’s correction, using GraphPad Prism. **** represents p < 0.0001 (**B**) Labile iron pool, “LIP”, was quantified in iron overload HepG2 cell lysates. Ascorbic acid concentrations in the working solution were varied from 10 mM to 1 M. In LIP measurements, there was a significant increase in the detection of labile iron when using 250 mM (p < 0.0001) and 1 M (p = 0.0080). (**C**) Total iron was quantified in the same cell lysates after digestion with nitric acid. Ascorbic acid concentrations also varied from 10 mM to 1 M. In total iron measurements, there was a significant increase in iron concentration when using 250 mM (p = 0.0421) and 1 M (p < 0.0001) ascorbic acid. One-way ANOVA was performed with Dunnett’s correction using GraphPad Prism. (**D**) Total iron was quantified using both ICP-MS and the *u*-ferene (5 mM ferene and 1 M ascorbic acid) assay in unloaded and iron overloaded HepG2 cells. Similar results were obtained for both ICP-MS and the *u*-ferene assay; no significant difference is observed between either the control or the iron overload cells. All experiments were done in at least triplicates. Error bars show standard deviations. Statistical analyses were performed using GraphPad Prism.

In order to measure iron using the ferene assay, Fe(III) should be reduced to Fe(II), typically by ascorbic acid, followed by the bidentate chelation of Fe(II) by ferene to form a stable blue complex between pH 3 to 6, with a molar absorptivity of 34,500 L cm^−1^ mol^−1^^57^. In addition to ascorbic acid being a potent reducing agent, our working hypothesis was that the concentration of ascorbic acid has strong influence on the decomplexation iron complexes in the analyte (e.g. DFO-Fe). For this purpose, we used DFO, a hexadentate iron (III) chelator, with a pFe^3+^ of 26 to illustrate the significance of ascorbic acid in iron content measurement by the ferene assay^58–60^. We first optimized the ascorbic acid concentration in both buffer and in vitro conditions. Iron was measured in three analytes—100 µM of free iron (Fe), 2000 µM DFO only (DFO), and 100 µM iron pre-chelated with 2000 µM DFO (DFO-Fe)—using a working solution composed of 5 mM ferene in ammonium acetate buffer and varying concentrations (0–250 mM) of ascorbic acid (Fig. 1A).

At concentrations of ascorbic acid lower than 10 mM, there is a significant difference between iron concentrations measured in Fe analyte and DFO-Fe analyte, p < 0.0001 at all concentrations. In other words, there is a significant difference between the iron measured in the two analytes. Interestingly, at ascorbic acid concentrations of 25 mM and above, the iron concentrations measured by ferene in free iron (Fe) and DFO-Fe are not significantly different. DFO only samples show no detection of iron, as expected. This data illustrates that concentration of ascorbic acid has a strong role on the removal of iron from Fe-DFO complex. This observation is of particular interest because it warrants further investigation into the utility of ascorbic acid concentration to discern between labile and total iron with the biological context—labile iron is redox active iron within the cells and total iron is all iron, both redox active and inactive.

### The role of ascorbic acid in labile and total iron content measurement by the ferene assay

Next, the role of ascorbic acid was investigated to distinguish the measurement of labile and total iron from cell lysates. HepG2 cells were iron loaded with iron and lysates were prepared. Labile and total iron was measured in cell lysates before (Fig. 1B) and after (Fig. 1C) nitric acid digestion. Four different working solutions were used to measure iron content, consisting of 5 mM ferene and varying ascorbic acid concentrations (10, 50, 250 and 1000 mM). It is important to highlight two aspects. First, only iron that is chelated by ferene enables iron measurements and that ferene chelatable iron is dependent on the ascorbic acid concentrations present in the working solution. Second, cellular iron concentrations are reported as a ratio of iron concentration to protein concentration rather than iron concentration alone^61–63^ or relative to the number of cells^21,64–67^. We anticipate that protein concentrations better account for fluctuations associated with handling of cells and reflect changes due to cell death.

In iron overload cell lysates without nitric acid digestion, no difference is observed in the ferene-based iron detection from working solutions with 10 mM or 50 mM ascorbic acid. However, significantly more iron was quantified as ascorbic acid concentration increased from 50 to 250 mM and then from 250 to 1000 mM (p < 0.0001 and p = 0.0080, respectively). This increase is suggestive of iron being released from iron complexes in the undigested cell lysates.

Accounting for the observations made from Fig. 1A, 10 mM of ascorbic acid yields a significant difference in iron detection when comparing free iron and pre-chelated DFO iron, while not being significantly different in iron overloaded cell lysates. Therefore, we can define labile iron as iron measured in undigested samples by 5 mM ferene using a working solution with 10 mM ascorbic acid. The labile iron concentration determined represents the labile iron from various intracellular compartments.

Figure 1C shows the iron determined in iron overload cell lysates that underwent nitric acid digestion. The total iron concentrations determined were significantly increased when comparing working solutions with 50 mM and 250 mM ascorbic acid, and again when comparing 250 mM and 1000 mM ascorbic acid (p = 0.0421 and p < 0.0001). Based on this data, we defined the total iron as iron measured in acid-digested samples by 5 mM ferene using a working solution with 1 M ascorbic acid. The requirement for high ascorbic acid concentrations is likely due to two reason; first, more iron is available after acid-digestion, and second, more of this iron is present in the ferric form which needs to be reduced. This is further corroborated by ICP-MS analysis of these samples, which shows no significant differences in the total iron measurements (Fig. 1D).

### Distinguishing “labile” and “chelatable” iron using a 2-in-1 *u*-ferene assay

**Figure 2:**
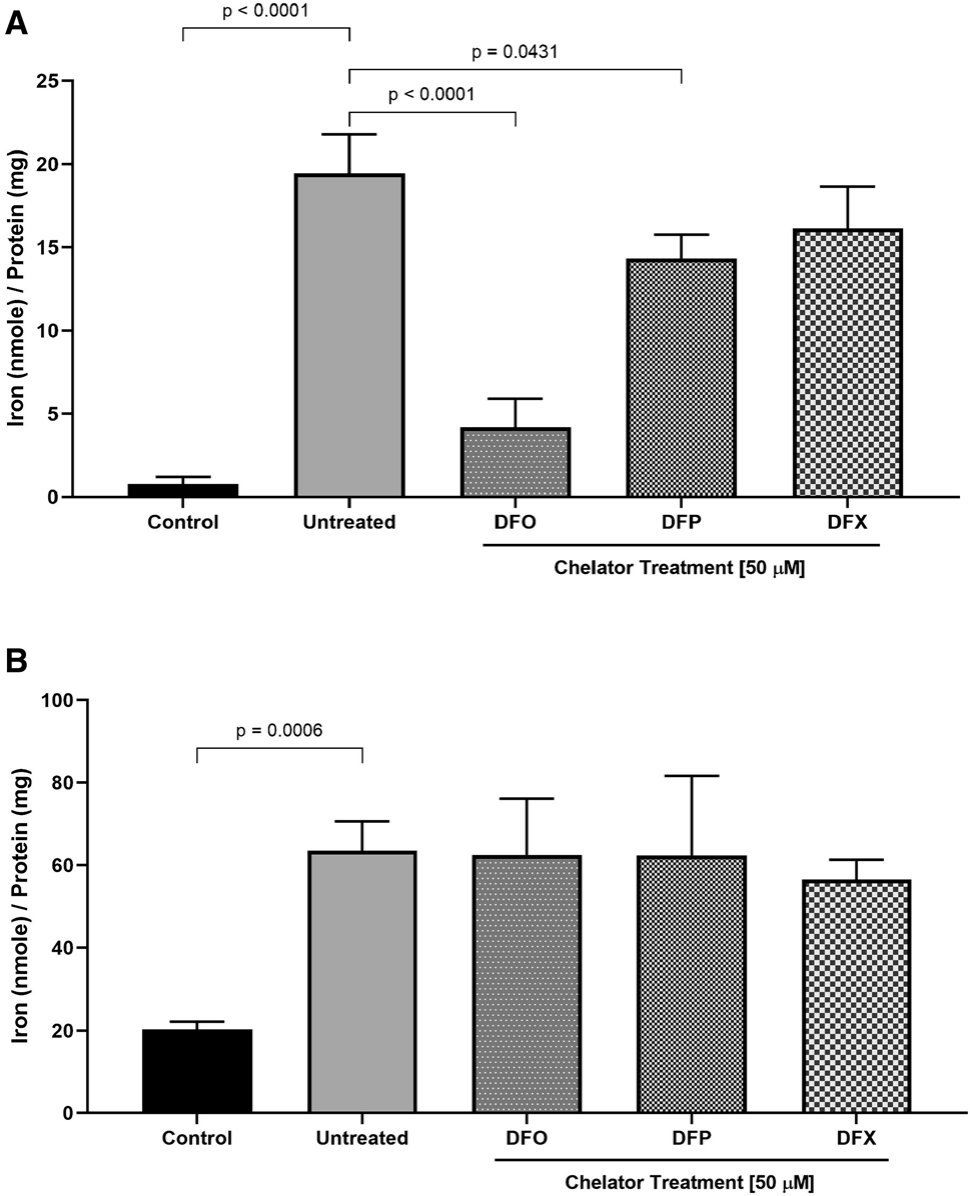
The confirmation of measuring chelatable labile iron in vitro. HepG2 cells were loaded with 200 μM of iron using FAC. Cells were lysed and these lysates were treated with either deferoxamine (DFO), deferiprone (DFP) and deferasirox (DFX) at 50 μM overnight. (**A**) LIP—measured using 5 mM ferene and 10 mM ascorbic acid in lysates. Chelatable labile iron were measured in cell lysates with iron chelator treatments. Labile iron was measured in cell lysates without iron chelator treatments. (**B**) Total iron—measured in nitric acid digested cell lysates followed by the ferene-based assay for total iron (5 mM ferene and 1 M ascorbic acid). One-way ANOVA was performed with Tukey’s correction using GraphPad Prism.

Building on the previous observation, we next sought to validate iron measurements using our developed 2-in-1 *u*-ferene assay. To determine the labile iron pool (LIP) and chelatable labile iron, undigested iron overload HepG2 cells lysates were prepared with and without treatment of clinically approved iron chelators—deferoxamine (DFO), deferiprone (DFP) and deferasirox (DFX)—at 50 µM. Iron concentrations were measured in both sets of samples and are shown in Fig. 2. Chelatable labile iron is defined as the portion of the labile iron that is chelated by the iron chelators. DFO and DFP treatment displayed a significant decrease in labile iron when compared to the untreated iron overloaded cell lysates (p < 0.0001 and p = 0.0431, respectively) (Fig. 2A). Total iron was measured using the total iron protocol and, expectedly, no significant changes were observed (Fig. 2B). Further, there is reproducibility of the total iron content with previous experiments, as shown in Fig. 1C, and ICP-MS, Fig. 1D. Of great importance, the data speaks to the importance of ascorbic acid concentration to differentiate between total and labile iron, as well as chelatable iron with respect to different chelators (Supplementary Fig. S3).

### Quantification of total iron in various tissue specimens using the *u*-ferene assay

**Figure 3:**
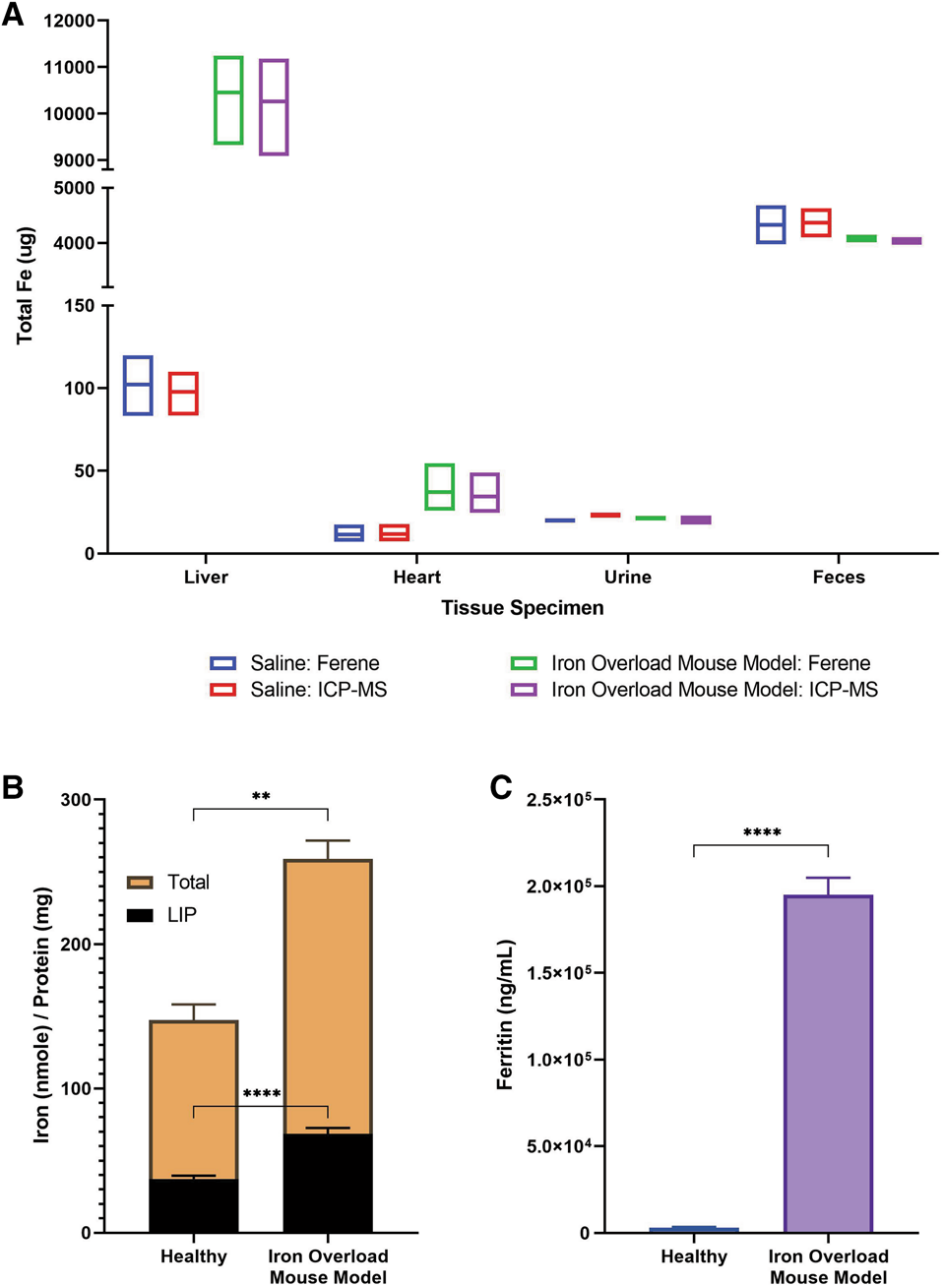
Quantification of total iron in various tissue specimens; organs (liver and heart), fluids (urine and plasma), and solid excrements (feces). (**A**) Total iron was measured in both saline (red and blue) and iron-dextran iron overload mouse model groups (green and purple) comparing the ferene assay (blue and green) to ICP-MS (red and purple). The total iron measurements used 5 mM ferene and 1 M ascorbic acid as the working solution in the *u*-ferene assay. (**B**) Plasma labile iron (5 mM ferene and 10 mM ascorbic acid) and total iron (5 mM ferene and 1 M ascorbic acid) were measured from healthy and iron overloaded mice using the ferene assay. (**C**) Plasma ferritin concentrations were measured using an ELISA assay from health and iron overload mice. Three biological replicates were measured per group. Unpaired t-test were performed using GraphPad Prism. **** represents p < 0.0001 and ** represents p < 0.01.

Total iron content from tissue specimens are shown in Fig. 3. The tissue samples were from iron overloaded mice developed by injecting iron-dextran^52,53^. Iron concentrations determined either via ICP-MS and the *u*-ferene assay from different tissue samples are almost identical (Fig. 3A), irrespective of the complexities associated with tissue specimens, which confirms the accuracy and utility of this benchtop technique. In the contest of this experiment, accuracy of the *u*-ferene assay refers to the comparison of the iron concentrations determined from ICP-MS since ICP-MS is the current gold standard for elemental analysis^18^. In addition, like ICP-MS, the *u*-ferene assay is able to measure a wide range of iron concentrations—from 10 μg iron in the whole heart to 10.300 mg iron in the whole liver (1030-fold range of concentration).

Furthermore, we applied the *u*-ferene assay to plasma specimens as well; significantly higher concentrations of both labile and total plasma iron (p < 0.0001 and p = 0.0055, respectively) than healthy mice controls were observed (Fig. 3B). The increase in total plasma iron was further corroborated by the significant elevation in ferritin concentrations (p < 0.0001) (Fig. 3C). This demonstrates the potential of using this convenient assay to investigate differences in labile plasma iron (and *non-transferrin bound iron (NTBI))* in various disease states.

## Discussion

Robust and accessible methods are required to measure iron levels, both labile and total iron, in different tissue specimens. Current approaches to quantitate iron exist, however they require specialised methodologies, extensive sample preparation and costly equipment^13–23^. Colorimetric methods provide an accessible, economic and are routinely refined for better sensitivities, convenient sample preparation and robust applications. Several chromogenic substrates, specific for chelatable ferrous iron, absorb strongly in the UV/Vis region, including bathophenanthroline sulfonate, ferrozine or ferene^17,21,25,26,68–70^. Reimer et al*.* developed a common assay for quantification of iron in astrocytes, using permanganate-mediated digestion and ferrozine-based colorimetric iron determination^17^. Hedayati et al*.* then improved Reimer et al*.* assay’s sensitivity by using nitric acid-mediated digestion and ferene-based colorimetric iron determination^21^. Ferene binds iron optimally between pH 3 to 6, with pFe^2+^ of 15^57^. However, the quantification of both labile and total iron from the same biological samples is yet to be reported using a ferene assay. It is worthwhile to mention that the quantification of labile iron pools are often tied to the specific fluorescent sensor, environmental variations within cells, and solvent systems used for calibrations^47^. As such, it is difficult to directly translate these concentrations to other modes of analysis.

The labile iron pool is critical for cellular iron metabolism and exists in dynamic equilibrium between different organelles^3,11,71^. Current methods to trace and quantitate labile iron concentration measure changes in fluorescence from iron sensors, which either turn-on (fluorescence enhances) and turn-off (fluorescence quenches) in the presence of iron^11,31^. These fluorescence techniques to measure metals is a rapidly growing approach offering spatial and temporal resolution. However, the use of fluorescence sensors are greatly hindered by limited commercially available probes, poor metal selectivity, mediocre signal-to-noise ratio, and requires specialized instruments (fluorescence microscopy or flow cytometry)^11,31^. Taken together, this undermines the absolute quantification of the labile iron pool.

In this investigation, we report an easy-to-use ferene-based protocol, *u*-ferene assay, that enables a 2-in-1 detection of both total and labile iron in biological samples, by varying ascorbic acid concentrations. Ascorbic acid is one of the low-molecular ligands that reduces ferric iron to ferrous iron, which then enables ferene, a bidentate chelator with a pFe^2+^ of 15, to chelate ferrous iron^6,57,72^. Moreover, this reducing agent also has the potential to interrupt stably chelated iron. To assess the ferene-based detection of iron, iron pre-chelated with DFO, was used. We used DFO as our model iron chelator because it has been extensively investigated and offers a particular advantage due to its hexacoordinate sphere with iron and high affinity (pFe^3+^ of 26)^58–60^. Based on our preliminary screening experiments, it is confirmed that ascorbic acid concentration has a central role in these measurements; significant release of iron from DFO-Fe coordination sphere was observed with the concentration of ascorbic acid above 10 mM (Fig. 1A). In other words, ascorbic acid above 10 mM is sufficient to reduce all iron pre-chelated with DFO. Therefore, we chose 10 mM ascorbic concentration for the determination of labile iron. This is because the iron measured at 10 mM ascorbic concentration sufficiently discerns between the iron only analyte and the iron pre-chelated with DFO analyte (p < 0.0001). It is important to note that these working concentrations may change when using other iron chelators. Within a biological context, DFO bound iron serves as an indicator for stably bound iron, and at 10 mM ascorbic acid, we can conservatively discern between chelatable and unchelatable iron.

In this report, we defined labile iron as redox active iron quantified using 5 mM ferene and 10 mM ascorbic acid from a biological sample. The labile iron concentrations in non-iron loaded HepG2 cells were found to be 0.8 nmole ± 0.2 nmole/mg of protein (4 μM) (Fig. 2A) by the *u*-ferene assay. This is consistent with the reported values using fluorescent based labile iron determination methods. It has been reported that there is an average of 2 to 5 μM (equivalent to 0.3 to 0.9 nmole) of intracellular chelatable iron within normal rat hepatocytes^46,47,73^. Moreover, the increase in labile iron after iron loading HepG2 cells was also shown using calcein as a metallosensor for intracellular labile iron (Supplementary Fig. S2). While there is agreement in the labile iron concentrations determined by two different techniques in two different hepatocyte preparations, it is important to note that these diversities pose a challenge to validate labile iron concentrations thoroughly.

On the other hand, total iron is defined as all iron, redox active and unchelatable stored iron, quantified using 5 mM ferene and 1 M ascorbic acid after nitric acid digestion in a biological sample. We noticed 10 mM ascorbic acid concentration is not enough to completely reduce the iron after acid digestion of cell lysates (Fig. 1C) and ferene-based iron detection increased with ascorbic acid concentration. We hypothesize that this is due to the equilibrium between Fe^3+^ and Fe^2+^ under acidic conditions requiring more ascorbic acid to reduce the iron, since all the stable and otherwise unchelated iron have been released via nitric acid digestion. At 1000 mM ascorbic acid concentration, the total iron measurements agreed with ICP-MS analysis (Fig. 1D). Thus, by optimizing for ascorbic acid concentration needed to sufficiently reduce iron for the detection by ferene, we were able to quantitate both labile iron and total iron (Fig. 1).

To further validate this assay’s capacity to quantify chelatable iron in cell lysates, the labile iron pool was perturbed by the introduction of high-affinity iron (III) chelators with stability constants above log(20) and the iron content was determined by the *u*-ferene assay (Fig. 2A). The monitoring of different cellular iron pools is of utmost importance, especially within the field of hereditary and transfusion-dependent iron overload, since the production of reactive oxygen species has been correlated to changes in the labile iron pool^74,75^. Cell lysates were used for two reasons; first, it ensured changes in the labile iron pool without affecting the total iron and second, it demonstrates the use of the same lysate for both labile and total iron determination. Upon treatment with DFO and DFP, the labile iron pool is significantly lowered because these chelators strongly bind to the intracellular ferric iron hence reducing the measurable labile iron pool (Fig. 2A). DFX treatment shows only a slight decrease in the labile iron pool. This may speak to the tunability of this assay, with respect to ascorbic acid concentration, for other chelators (Supplementary Fig. S3). Of note, total iron concentration is not different across treatments since only the lysates were manipulated.

Next, total iron was quantified using the same *u*-ferene assay with two modifications; first, biological samples underwent nitric acid digestion to release all iron^21^ and second, 1 M ascorbic acid concentration in the working solution was used. Total iron concentrations loaded within HepG2 cells were in the range of 3.5 to 5.0 μg iron per mg protein (70 to 90 nmole iron per mg protein across the different iron loading concentrations), which has been validated by ICP-MS (Fig. 1D) and is in agreement with earlier reports further confirming the utility of our assay^76–78^. Moreover, the total iron content did not change despite increasing iron loading concentrations. This pattern of iron accumulation has been observed to be unique in HepG2 cells (Fig. 2B)^78^.

To further explore the robustness of the *u*-ferene assay, we determined iron content from various biological specimens collected from mice (Fig. 3 & Supplementary Fig. S4) and compared the data with ICP-MS. For the sake of clarity, we have loosely categorised the different biological specimens in three groups; fluids (plasma and urine), organs (liver, spleen, kidneys, and heart) and solid excrement (feces). The total iron determined from the *u*-ferene assay is almost identical to that determined from ICP-MS in all biological specimens (Fig. 3)—the precision (i.e. standard deviations) of the *u*-ferene assay parallels that of ICP-MS. This also highlights the sensitivity of the *u*-ferene assay by offering a wide range of concentrations of iron detection (0 to 1000 μM), principally through acid-cleaned vials, sample manipulation and appropriate dilutions.

In addition, plasma iron from both groups were quantified with respect to labile and total iron. Both labile and total iron measurements were significantly elevated in the iron overload group when compared to the saline group (p < 0.0001 and p = 0.0055, respectively). This increase in total iron was further supported by the increase in ferritin concentration, as measured by the ferritin ELISA kit. Plasma, serum and blood specimens are readily tested clinical samples, and speak to potential adaptability of this assay. To further explore that source of the labile iron measured by the *u*-ferene assay and to determine whether the experimental conditions used in labile iron pool measurements could remove iron from proteins, in vitro experiments were performed using holo-transferrin (holo-Tf) (Supplementary Fig. S6). This suggests that some iron can be stripped and measured from proteins, like transferrin, using a working solution for labile iron measurements (5 mM ferene and 10 mM ascorbic acid). This might explain the elevated labile iron measured (a combination of potentially chelatable free iron (non-transferrin bound iron (NTBI)) and some additional iron released from proteins) in the healthy control (Fig. 3A). On the other hand, the working solution for total iron measurements (5 mM ferene and 1 M ascorbic acid) in concert with nitric acid digestion is sufficient to measure all iron since the *u*-ferene assay measures the same concentration of iron as provided by Sigma Aldrich's certificate of analysis.

Taken all together, using our *u*-ferene colorimetric benchtop assays enables the quantification of iron—*both labile and total iron*—in a variety of biological specimens. This assay offers the precision and sensitivity similar to that of ICP-MS and fluorescent based methods with the added cost-saving and readily accessible methodology.

## Conclusions

We reported the development of an economic and readily accessible alternative assay for the measurement of both labile and total iron using the unified-ferene (*u*-ferene) assay, whilst maintaining the accuracy and sensitivity as compared to the current gold standard. This assay enables a 2-in-1 quantification of iron from the same preparation of a biological specimen, circumventing tedious, expensive and laborious methodologies. We demonstrated that labile iron can be distinguished from total iron using different ascorbic acid concentrations in the working solutions as well as performing acid-digestion on samples. This method provides adequate quantification of both chelatable and labile iron as well. Iron quantification, both labile and total iron, agree with literature as well as comparison with other currently available techniques. Quantification of iron is a necessity to assess the iron burden in patients suffering from iron overload diseases, as well as tremendous value in understanding the underlying role of iron in the pathophysiology of these diseases. Thus, the *u*-ferene assay brings considerable value for the wider scientific community.

## Supplementary information

Supplementary information.

## Data Availability

All data is available in the main text or the supplementary materials.
