# Supplementary material for: A facile colorimetric method for the quantification of labile iron pool and total iron in cells and tissue specimens
Source: Sci Rep. 2021 Mar 16;11:6008. doi: 10.1038/s41598-021-85387-z (PMC7971025; doi:10.1038/s41598-021-85387-z)
Supplement: Supplementary file 1 — Supplementary information. [file 41598_2021_85387_MOESM1_ESM.pdf]

A Facile Colorimetric Method for the Quantification of Labile Iron Pool and Total Iron in Cells and Tissue Specimens

Usama Abbasi,[1,2,#] Srinivas Abbina,[1,2,#] Arshdeep Gill,[1,2,3] Vriti Bhagat,[2] Jayachandran N. Kizhakkedathu[1,2,3,4,*]

[1]Department of Pathology and Laboratory Medicine, The University of British Columbia, Vancouver, BC, Canada.
[2]Centre for Blood Research, Life Sciences Institute, The University of British Columbia, Vancouver, BC, Canada.
[3]Department of Chemistry, The University of British Columbia, Vancouver, BC, Canada
[4]The School of Biomedical Engineering, The University of British Columbia, Vancouver, BC, Canada

[#]equally contributed
[*]address to which correspondence to be addressed.

Supplementary Figures and Tables:

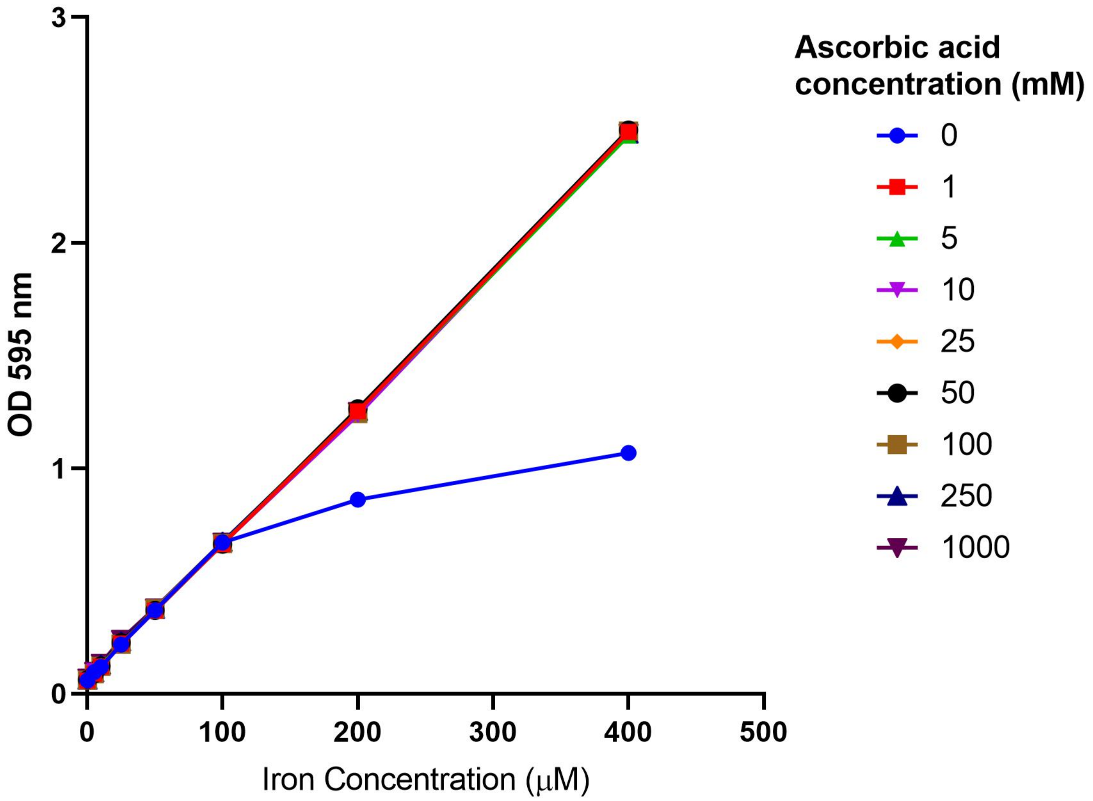

Supplementary Figure S1. The standard curve for the ferene assay with different concentrations of ascorbic acid in the working solution. While ascorbic acid concentrations is critical for the differentiation of labile iron and total iron measurements, the standard curve was prepared under different ascorbic acid concentrations (mM). The linear regression for these three iron standard curves are not different. All experiments were done in at least triplicates. Error bars show standard deviations. Statistical analysis were performed using GraphPad Prism.

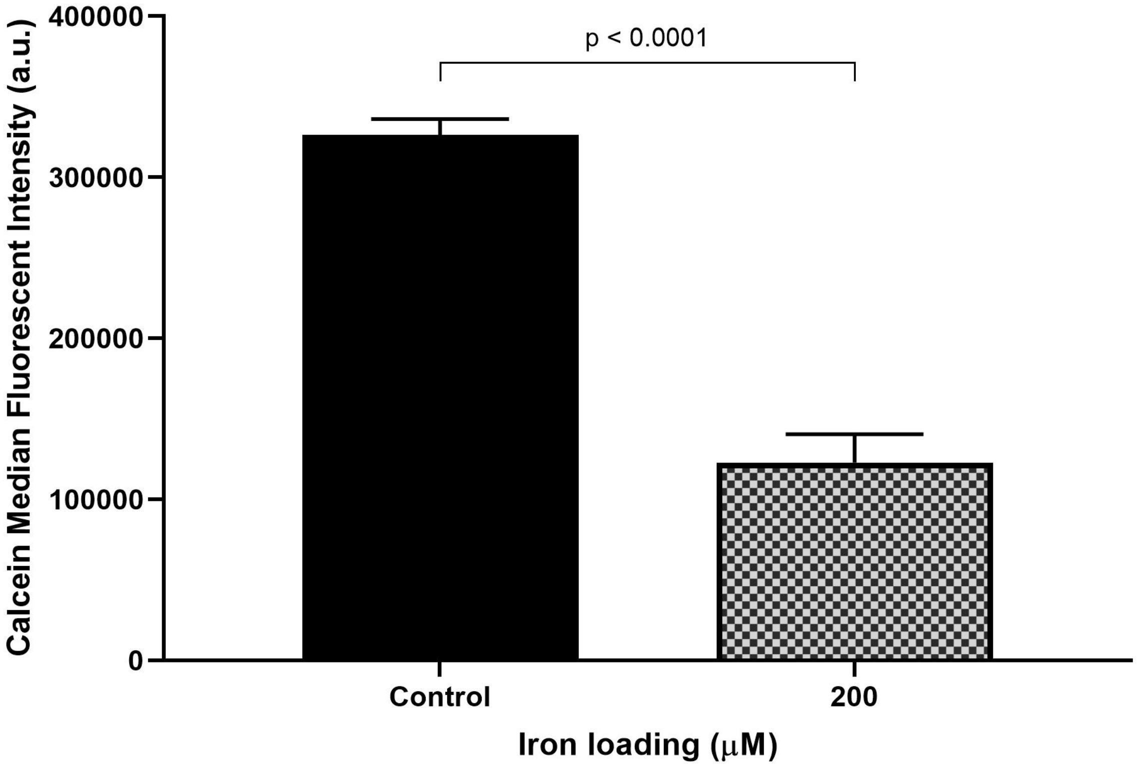

Supplementary Figure S2. The effect of iron loading on labile iron concentration. Calcein is a fluorophore which quenches in the presence of iron. This fluorophore was used to monitor the changes in the intracellular labile iron pool using flow cytometry. Unpaired t-test was performed using GraphPad Prism. All experiments were done in at least triplicates. Error bars show standard deviations. Statistical analysis were performed using GraphPad Prism.

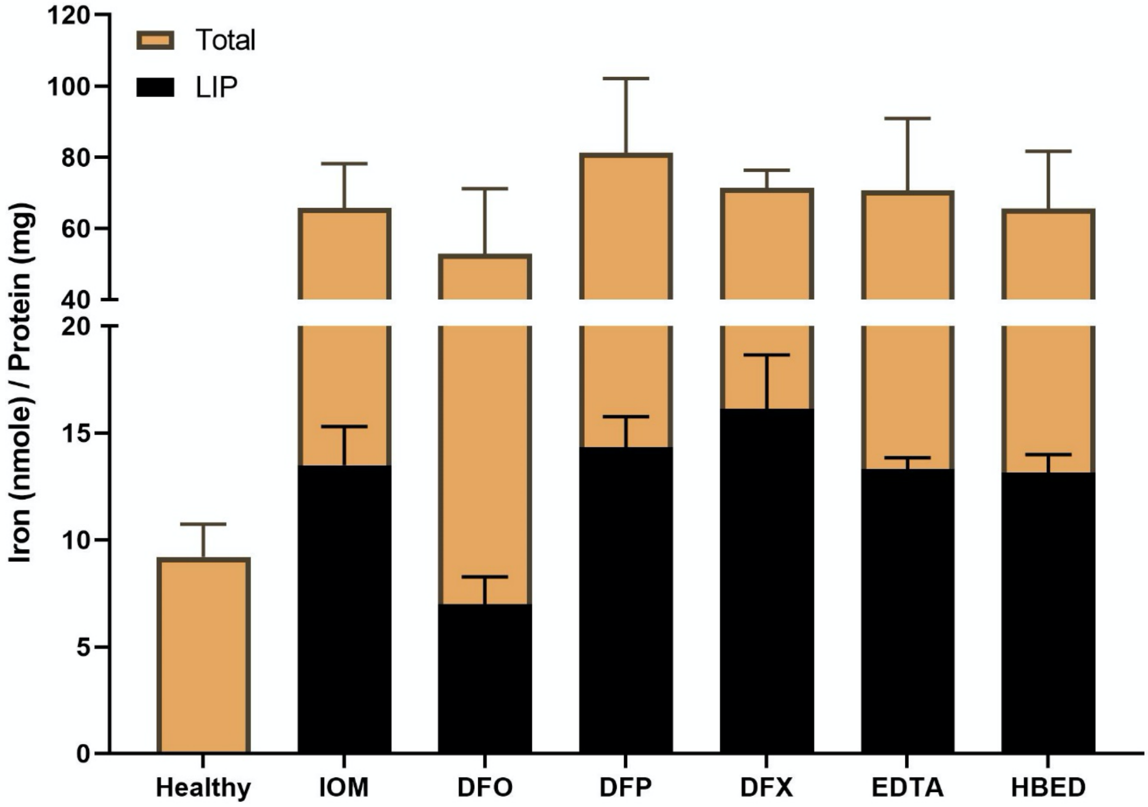

Supplementary Figure S3. Changes in the labile iron pool (LIP) in the presence of different chelators. LIP (using 10 mM ascorbic acid in the working solution) and total iron was quantified from iron overloaded HepG2 cell lysates; IOM – iron overload model with no chelator, DFO – deferoxamine, DFP – Deferiprone, DFX – Deferasirox, EDTA - Ethylenediaminetetraacetic acid, and HBED - N, N-bis(2-hydroxybenzyl)ethylenediamine-N,N-diacetic acid. Healthy cell lysates were used a control for changes in LIP and total iron. At 10 mM ascorbic acid concentration in working solution, only DFO treated lysates had a significant reduction in the LIP ($p<0.0001$). All experiments were done in at least triplicates. Error bars show standard deviations. Statistical analysis were performed using GraphPad Prism.

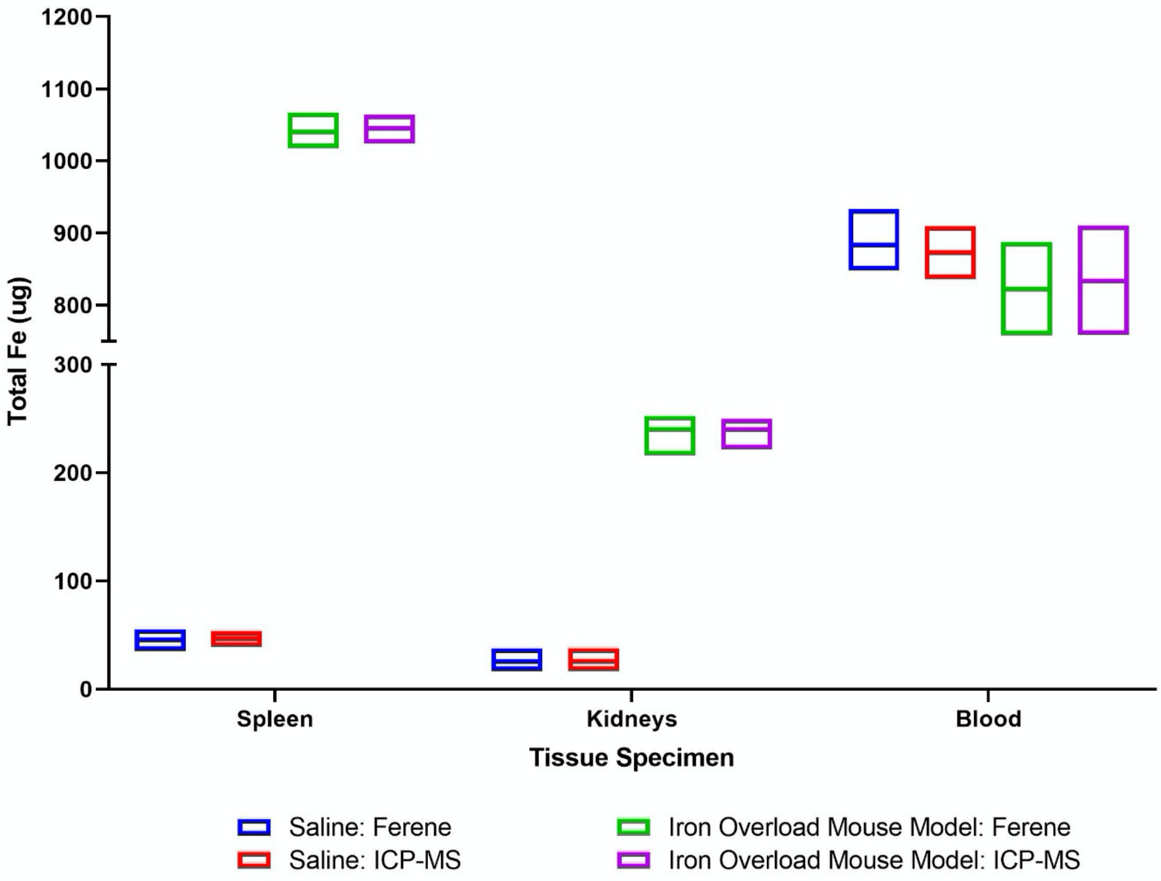

Supplementary Figure S4. Quantification of total iron in the remaining tissue specimens; organs (spleen and kidneys) and fluids (blood). Total iron was measured in both saline (red and blue) and iron-dextran iron overload mouse model groups (green and purple) comparing the ferene assay (blue and green) to ICP-MS (red and purple). Three biological replicates were measured per group. Statistical analysis were performed using GraphPad Prism.

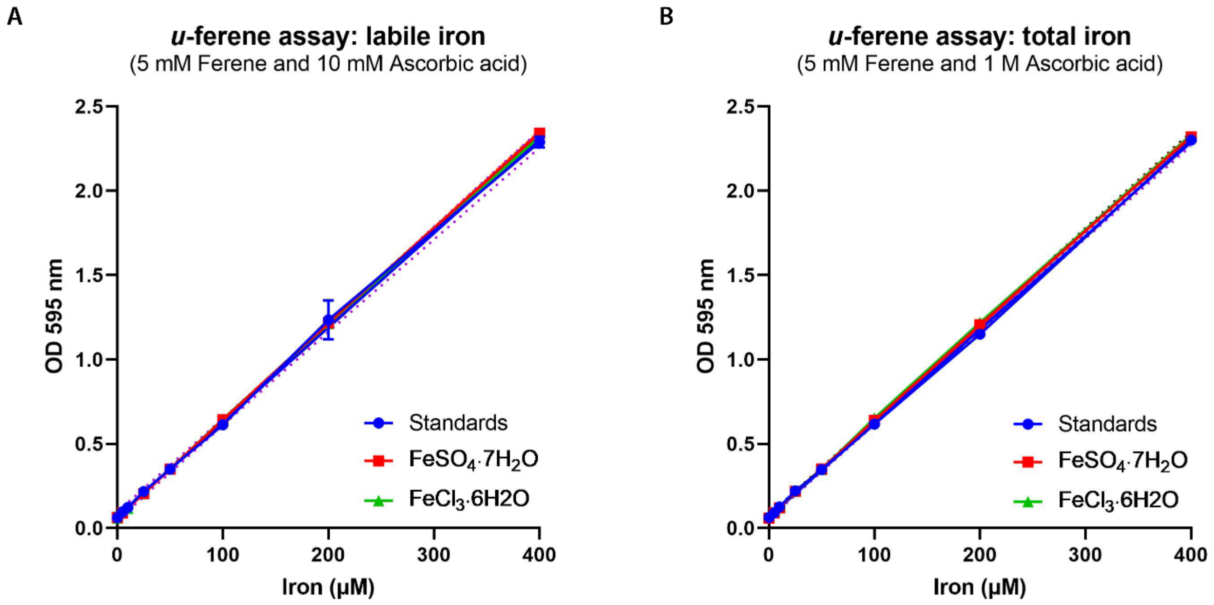

Supplementary Figure S5. The standard curve for the ferene assay with different source of primary iron sources – ferric chloride and ferrous sulphate. Iron standards were prepared from either ferric chloride or ferrous sulphate, ranging from 0 to 1000 μM. The standard curve was generated using the experimental conditions defined for labile iron measurements (5 mM ferene and 10 mM ascorbic acid) and total iron measurements (5 mM ferene and 1 M ascorbic acid). All experiments were done in at least triplicates. Error bars show standard deviations. Simple linear regressions and their respective 95% confidence intervals were determined using GraphPad Prism. The slope and y-intercept determined from the linear regression were not significantly different amongst the different sources of iron, as determined by the overlap in the 95% confidence intervals.

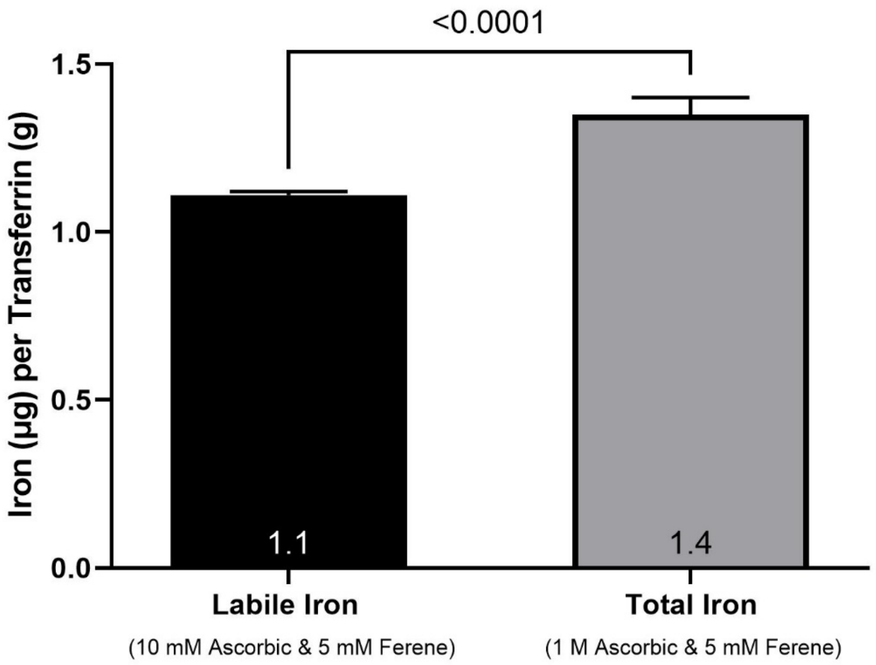

Supplementary Figure S6. Labile and total iron were determined using the *u*-ferene assay in holo-transferrin. All experiments were done in at least triplicates. Error bars show standard deviations. Unpaired t-test was performed to compare labile and total iron measurements. One sample t test was performed to compare total iron measurements with the iron content determined in the certificate of analysis from Sigma Aldrich. All statistical analyses were performed using GraphPad Prism.

Supplementary Table 1. A list of dilutions used for the analysis of total iron in biological samples

biological sample	dilution for *u*-ferene	dilution for ICP-MS
cell lysates	1	3
plasma	1	3
liver	40	5000
spleen	20	1000
heart	1	40
kidneys	8	250
urine	1	10
feces	80	4000
blood	10	1500

Supplementary Table 2. The template of running assay for the determination of iron content

		working solution (5 mM ferene in ammonium acetate buffer) with varying ascorbic acid concentrations (0-1000 μM)								
		0	1	5	10	25	50	100	250	1000
Analytes	Fe									
	Fe-DFO									
	DFO									
iron standards (μM)	0									
	5									
	10									
	25									
	50									
	100									
	200									
	400									

Supplementary Table 3. The main components in the *u*-ferene assay's working solution

working solution components	labile iron	total iron
Ferene	5 mM	5 mM
Ascorbic acid	10 mM	1 M
Prepared in ammonium acetate buffer (pH 4.5, 2.5 M)		
